# Mesoscopic chaos mediated by Drude electron-hole plasma in silicon optomechanical oscillators

**DOI:** 10.1038/ncomms15570

**Published:** 2017-06-09

**Authors:** Jiagui Wu, Shu-Wei Huang, Yongjun Huang, Hao Zhou, Jinghui Yang, Jia-Ming Liu, Mingbin Yu, Guoqiang Lo, Dim-Lee Kwong, Shukai Duan, Chee Wei Wong

**Affiliations:** 1 College of Electronic and Information Engineering, Southwest University, Chongqing 400715, China; 2 Fang Lu Mesoscopic Optics and Quantum Electronics Laboratory, University of California, Los Angeles, California 90095, USA; 3 Electrical Engineering, University of California Los Angeles, California 90095, USA; 4 Institute of Microelectronics, A*STAR, Singapore 117865, Singapore

## Abstract

Chaos has revolutionized the field of nonlinear science and stimulated foundational studies from neural networks, extreme event statistics, to physics of electron transport. Recent studies in cavity optomechanics provide a new platform to uncover quintessential architectures of chaos generation and the underlying physics. Here, we report the generation of dynamical chaos in silicon-based monolithic optomechanical oscillators, enabled by the strong and coupled nonlinearities of two-photon absorption induced Drude electron–hole plasma. Deterministic chaotic oscillation is achieved, and statistical and entropic characterization quantifies the chaos complexity at 60 fJ intracavity energies. The correlation dimension *D*_2_ is determined at 1.67 for the chaotic attractor, along with a maximal Lyapunov exponent rate of about 2.94 times the fundamental optomechanical oscillation for fast adjacent trajectory divergence. Nonlinear dynamical maps demonstrate the subharmonics, bifurcations and stable regimes, along with distinct transitional routes into chaos. This provides a CMOS-compatible and scalable architecture for understanding complex dynamics on the mesoscopic scale.

## Abstract (web summary)

Chaotic behaviour of optomechanical systems has only recently been investigated and observed. Here, Wu *et al*. study the chaos dynamics in a silicon platform where coupled electron-hole plasma dynamics is possible, providing a route towards chip-scale mesoscopic nonlinear dynamics.

Investigation of chaos and the associated nonlinear dynamics has spurred fundamental progress of science and technology. It brought new perspectives in a multitude of fields spanning from recurrent neural networks^1^, relativistic billiards-like electron transport^2^, fractal space and time^3^ to self-organization in the natural sciences^4^, amongst others. Chaos in optical systems has emerged and drawn much attention owing to its unique features and broad applications, including chaos-based synchronized secure optical communications^5^,^6^,^7^, high-performance light detection and range finding^8^ and ultrafast physical random bit generation^9^. Studies of chaos generation in III–V laser components have further shown progress in harnessing the broadband carriers in both the near infrared and the mid-infrared wavelength ranges^10^,^11^,^12^,^13^,^14^,^15^,^16^,^17^, although the challenges of monolithic integration and circumventing the seemingly universal requirement of external perturbations remain to be solved.

Concurrently, significant efforts in nanofabrication technology and cavity optomechanics have led to the demonstration of regenerative oscillations in mesoscopic resonators^18^,^19^,^20^,^21^. Excited by centrifugal radiation pressure, optomechanical chaotic quivering was experimentally observed in toroidal whispering-gallery-mode microcavities^22^. Recently, in the toroidal whispering-gallery-mode microcavity, stochastic resonance and chaos have been transferred between two optical fields^23^ with the chaotic physical basis through a strong nonlinear optical Kerr response from the nonlinear coupling of the optical and mechanical modes. This is complemented by recent theoretical studies on chaos including electro-optomechanical systems and potential routes into chaos^24^,^25^.

Here, we couple the prior single optomechanical basis with a second basis—that of electron–hole plasma oscillations in the same cavity—to deterministically generate dynamical chaos in a silicon photonic crystal cavity. Differing from the prior studies, the silicon experimental platform enables electron–hole plasma dynamical generation, destabilizing the system dynamics and provides a route for chip-scale planar electronic–photonic integration. Our photonic crystal implementation is based on a slot-type optomechanical (OM) cavity with sub-wavelength [≈0.051(*λ*/*n*_air_)^3^] modal volumes *V*, and high quality factor-to-volume ratios *Q*/*V* (refs 26, 27). This provides strong optical gradient oscillation^26^,^28^ to achieve operating intracavity energies of ∼60 fJ and enables near-single-mode operation. Our two-oscillator OM cavity is designed with comparable dynamical oscillation timescales between the Drude electron–hole plasma and radiation pressure optomechanics, which allows the chaotic attractors and unique trajectories to be uncovered. We present the statistical and entropic characteristics of the nonlinear dynamical regimes and illustrate the transition routes into and out of chaos. Our first-principles numerical modelling, including coupled oscillations in seemingly unrelated degrees of freedom (two-photon-induced free-carrier and thermal dynamics with radiation pressure dynamics) capture the experimental observations, the multi-period orbits and the trajectory divergence into chaotic states.

## Results

### Experimental observation of chaos

**Figure 1:**
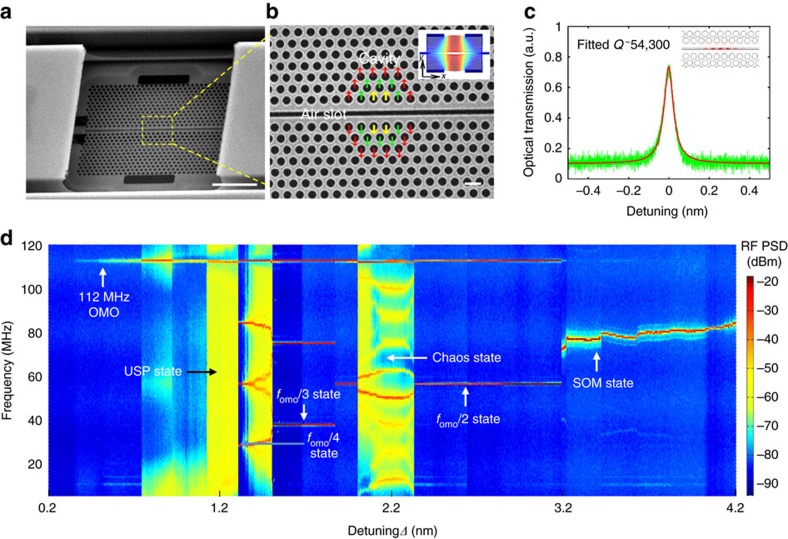
Observations of dynamical chaos in mesoscopic optomechanical cavities. (**a**) Scanning electron micrograph of the optomechanical cavity. Scale bar, 5 μm. (**b**) Zoom-in of 120 nm slot cavity with localized resonant mode formed by perturbed neighbouring holes at the cavity centre, with amplitude displacements denoted by the coloured arrows (yellow: 15 nm; green: 10 nm; and red: 5 nm). The lattice constant is 500 nm and the ratio between hole radius and lattice constant is 0.34. Scale bar, 500 nm. Inset: finite-element model of the fundamental mechanical mode field, with normalized displacement magnitude shown in colour (red as maximum displacement and blue as zero displacement). (**c**) Measured optical transmission spectrum with a cold cavity loaded quality factor *Q* of 54,300 under low injection power and centred at 1572.8 nm. Inset: |*E*|^2^ field distribution of the fundamental optical resonance, with normalized intensity magnitude shown in colour (red as maximum intensity and white as zero intensity). (**d**) 2D RF spectral map illustrating the evolution of nonlinear and chaotic dynamics, detailed as OMO (OMO) state - USP state-*f*_omo_/4 state-*f*_omo_/3 state-chaos state-*f*_omo_/2 state-SOM state, under controlled laser-cavity detuning *Δ* and at 1.26 mW injection power.

Figure 1a shows the scanning electron micrograph of the slot-type optomechanical photonic crystal cavity mediated by Drude electron–hole plasma investigated in this study. The air-bridged photonic crystal cavity is introduced with shifted-centre air holes that are shifted by 15, 10 and 5 nm, respectively, as shown in Fig. 1b. The width-modulated line-defect photonic crystal cavity design has a total quality factor *Q* of 54,300 (Fig. 1c) and a sub-wavelength modal volume of 0.051(*λ*/*n*_air_)^3^ (Fig. 1b inset) at the 1572.8 nm resonance wavelength (*λ*_o_, with effective mode index *n*). The optomechanical cavity consists of two (16.0 μm × 5.5 μm × 250 nm) micromechanical photonic crystal slabs, separated by a 120 nm slot width across the photonic crystal line defect. The in-plane mechanical mode has a 112 MHz fundamental resonance and, when driven into the regenerative oscillation regime, has a narrow sub-15-Hz linewidth at ambient pressure and room temperature^29^. The large optical field gradient from the tight slot cavity photon confinement enables a large coherent optomechanical coupling strength, *g*_0,_ of ∼690 kHz (detailed in Supplementary Note 4), resulting in low-threshold optomechanical oscillation (OMO)^26^,^27^,^28^,^29^. Concurrently, on the same cavity, strong nonlinearities such as two-photon absorption (TPA), free-carrier and thermo-optic dynamical effects lead to modulation of the intracavity field^30^. Note the characteristic timescales of the OMO and the photonic crystal carrier dynamics are made comparable through our designed mechanical modes and intrinsic free-carrier diffusion times, enabling the coupled equations of motion to have sufficient overlap and degrees of freedom for chaos generation.

Figure 1d depicts the transition into chaos as the pump detuning to the cavity resonance *Δ* (=*λ*_L_−*λ*_0_, where *λ*_L_ is the injection light wavelength) is scanned from 0.2 to 4.2 nm with the injection power fixed at 1.26 mW detailed in Methods section). The chaos region as well as the associated dynamical transitional states can be identified. First, a stable pure fundamental OMO at 112 MHz is observed at the beginning of the detuning drive. With increased detuning, aperiodic and sub-oscillatory structures emerges when *Δ* is set in the range of 1.2–2.0 nm. Unstable pulses (USP) occur first, before the system is driven into a series of stable sub-harmonic pulse states such as the *f*_omo_/4 states (oscillation period being four times the OMO period), the *f*_omo_/3 states and the *f*_omo_/2 states, respectively. For detuning *Δ* between 2.0 and 2.33 nm, the system exhibits a chaos region characterized by both a broadband radio frequency (RF) spectrum and an intricate phase portrait. For detuning *Δ*>2.33 nm, the system is driven to exit the chaos region by evolving into a *f*_omo_/2 state (*Δ*=2.33–3.2 nm) before cumulating into a self-induced optical modulation (SOM) state (*Δ*=3.2–4.2 nm)^30^,^31^. Of note, the oscillation period of SOM (∼13–17 ns), mainly determined by the Drude plasma effect and the thermal dissipation rate, is comparable with that of OMO (∼9 ns). The close oscillation frequencies of SOM and OMO facilitate their effective interaction in the photonic crystal nanocavity and the occurrence of chaos^4^,^18^.

**Figure 2:**
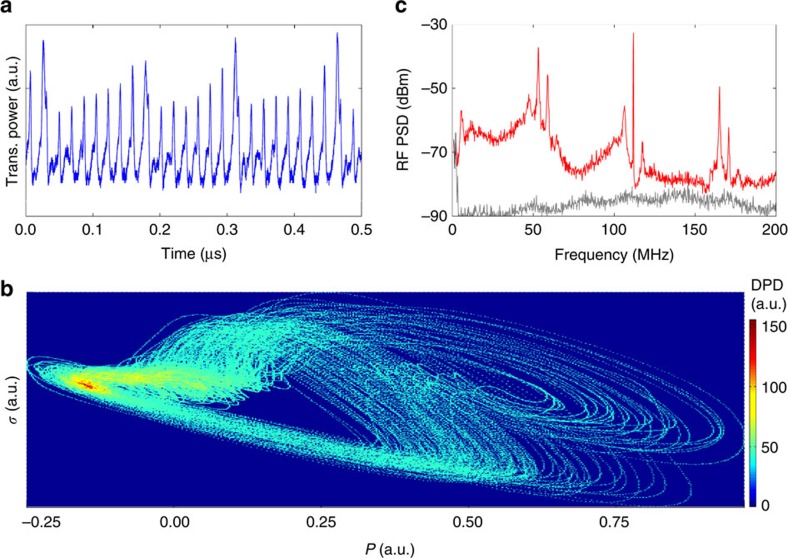
Frequency-time characterization of the chaos. (**a**) Raw temporal waveform of chaotic output. (**b**) Corresponding phase portraits of the noise-reduced temporal waveform, where the colour evolution from cyan to orange to red is proportional to the data point density (DPD) in the measured temporal orbit. (**c**) Corresponding measured RF power spectral density (PSD). The grey curve is the reference background noise floor.

Figure 2 shows an example chaotic oscillation in the temporal domain and its RF frequency spectrum with the recorded raw temporal waveform shown in Fig. 2a, illustrating the irregular and intricate fluctuations. Figure 2b presents the phase portrait of chaos in a two-dimensional plane spanned by the power of the temporal waveform (*P,* horizontal axis) and its first time derivative (*σ*, vertical axis)^32^. The reconstructed trajectory is useful for illustrating the complex geometrical and topological structure of the strange attractor, showing the local instability, yet global stable nature, of a chaos structure^32^. To reveal the topological structure of chaos attractors, a state-space procedure is implemented to average the temporal waveform points in an *m*-dimensional embedded space^32^ (detailed in Supplementary Note 1) by removing stochastic noise from the recorded raw data. The noise removal enables a clear depiction of the topological structure of the attractor and is also useful for the estimation of correlation dimension and Kolmogorov entropy, the most commonly used measures of the strangeness of chaotic attractors and the randomness of chaos^33^,^34^,^35^,^36^. Furthermore, Fig. 2c shows the corresponding RF spectrum, where the signal distributes broadly and extends up to the cutoff frequency of the measurement instrumentation, showing a hallmark spectral feature of chaos.

**Figure 3:**
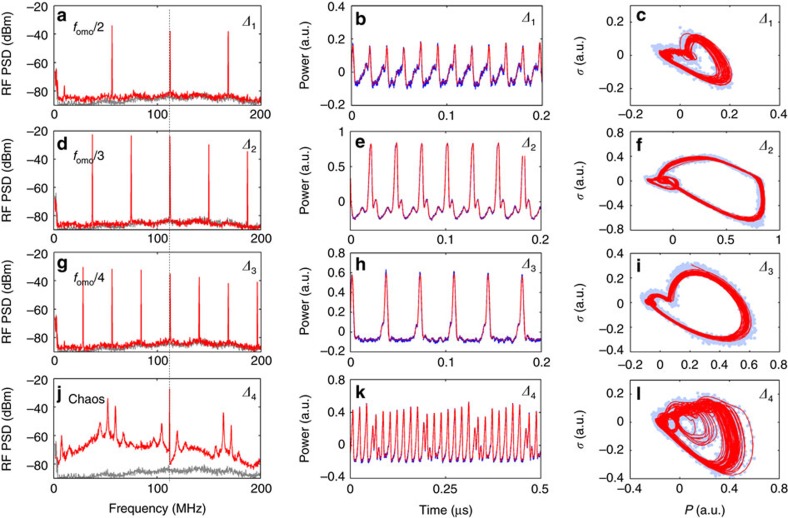
Dynamical states under controlled drive conditions. (**a**–**c**) the *f*_omo_*/*2 state (*Δ*_1_≈2.406 nm), (**d**–**f**) the *f*_omo_*/*3 state (*Δ*_2_≈1.831 nm), (**g**–**i**) the *f*_omo_*/*4 state (*Δ*_3_≈1.394 nm) and (**j**–**l**) the chaos state (*Δ*_4_≈2.285 nm) respectively. The curves (**a**,**d**,**g**,**j**) are the measured RF power spectral density (PSD) where the grey curves are the background noise floor. Notice the subharmonics have the background at the noise floor. The curves (**b**,**c**,**e**,**f**,**h**,**i**,**k**,**l**) are the temporal waveforms and orbital phase portraits, where the blue dots are the measured raw data and the solid red curves are the noise-reduced orbital trajectories.

Figure 3 illustrates the detailed properties of several different dynamical states, including RF spectra, temporal waveforms and phase portraits. First, Fig. 3a shows the frequency and temporal characteristics of the *f*_omo_/2 state. We observe three characteristic features of the *f*_omo_/2 state:distinct *f*_omo_/2 components in the RF spectrum (Fig. 3a), pulses with period (≈17.8 ns) at two times the OMO period (≈8.9 ns) in the temporal waveform (Fig. 3b), and clear limit cycle^37^ features in the phase portrait (Fig. 3c). Similarly, Fig. 3d–f,g–i show the frequency spectra, the temporal waveforms at a third and a quarter of the fundamental oscillation, and the corresponding limit cycle phase portraits of the transitional *f*_omo_/3 and *f*_omo_/4 states, respectively. We note the satellite bumps next to the main peaks in the temporal waveforms; they represent the relatively weak OMO fundamental oscillations. Figure 3j,k next show the frequency and temporal features of the chaos state, where a broadband spectrum and a fluctuating temporal waveform are observed. In the phase portrait (Fig. 3l), the trajectory evolves intricately and scatters widely in phase space, being quite different from other periodical dynamics. With this slot cavity and at 1.26 mW injection power (∼60 fJ intracavity energy), the specific transition route is OMO-USP-*f*_omo_/4-*f*_omo_/3-*f*_omo_/2-chaos-*f*_omo_/2-SOM, exhibiting a clear sub-harmonic route to chaos. The complete set of routing states into/out of chaos is detailed in Supplementary Note 2.

### Dynamical characterization of chaos

**Figure 4:**
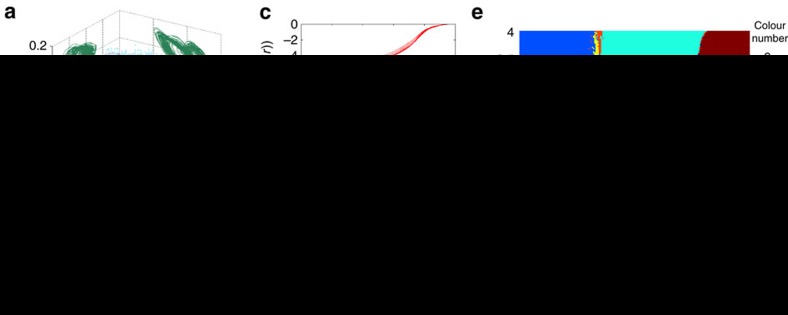
Chaos identification and regime distribution map. (**a**) Measured three-dimensional portrait in phase space. The blue dots are the measured raw data, while the solid red curve is the reconstructed trajectory. The three green phase portraits are projections of the 3D portrait onto the phase planes. (**b**) Calculated LEs spectrum. The curves converge to the LE values as *λ*_1_≈0.329, *λ*_2_≈−0.087 and *λ*_3_≈−0.946 ns^−1^. (**c**) Logarithmic plots of the correlation integral *C*_*D*_(*r*) versus sphere radius *r* based on the Grassberger-Procaccia algorithm. (**d**) Slope of the correlation integral versus sphere radius *r*. A clear plateau on the slope of the correlation integral is observed and marked with the horizontal dashed line. The correlation dimension *D*_2_ is estimated at ≈1.67. In **c**,**d**, the lines denote the vector size *D* from 15 to 20 in integer steps. (**e**) Dynamical distribution map based on the numerical modelling. Different colours denote different dynamical states, including OMO (OMO, dark blue) state, USP (light blue) state, *f*_omo_/3 state (yellow), *f*_omo_/2 state (cyan), chaos state (orange) and SOM (SOM, dark red) state. The OMO state, USP state, *f*_omo_/3 state and *f*_omo_/2 state denote the periodic and low-entropy dynamical regimes; the chaos state and SOM state denote the high-entropy dynamical regimes. The horizontal axis is the normalized laser-cavity detuning *δω*/γ_i_ and the vertical axis is the injected optical power *P*_in_. The dashed white horizontal line is an example corresponding to the injected power level in the measurement.

Next, statistical analysis is performed to uncover the detailed dynamical properties of the chaotic states. A three-dimensional phase space is constructed in Fig. 4a, in a volumetric space spanned by the power (*P*), the first time derivative of *P* (*σ*) and the second time derivative of *P* (*ξ*). The green curves are the projections of the trajectory onto each of the three phase planes, showing the geometric structures. Three statistical measures, Lyapunov exponents (LEs), correlation dimension and Kolmogorov entropy, are commonly employed to illustrate and characterize the dynamical properties of chaos^32^,^33^,^34^,^35^,^36^,^37^,^38^. Details of these measures are provided in Supplementary Note 1. LEs, which describe the divergence rate of nearby attractor trajectories, are the most widely employed criteria in defining chaos^33^. In Fig. 4b, we show the calculated LEs, converging to values *λ*_1_≈0.329, *λ*_2_≈−0.087 and *λ*_3_≈−0.946 ns^−1^ respectively, or equivalently, when expressed on the intrinsic optomechanical photonic crystal cavity, timescale (*τ*_omo_=*f*_omo_^−1^≈8.9 ns) *λ*_1_≈2.94*τ*_omo_^−1^, *λ*_2_≈−0.78*τ*_omo_^−1^ and *λ*_3_≈−8.45*τ*_omo_^−1^. The maximal LE is positive, illustrating a fast divergence rate between adjacent orbits and indicating that the system is chaotic^32^,^33^. We further analyse the correlation dimension *D*_2_:


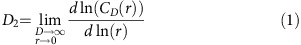


where *C*_*D*_ is the correlation integral of vector size *D* in an *r* radius sphere and *d* is the Euclidian norm distance^36^. A conservative estimate of the attractor correlation dimension is implemented through the Grassberger-Procaccia algorithm^36^,^38^ as detailed in Supplementary Note 1. As shown in Fig. 4c, the correlation integrals *C*_*D*_ vary with sphere radius *r*. In Fig. 4d, the plot of the correlation integral slope versus sphere radius *r* is obtained by extracting the slope from Fig. 4c. A clear plateau of the correlation integral slope is observed, supporting the estimated value of *D*_2_ at ∼1.67 (*D*_2_≈2.0 without noise filtering). The correlation dimension *D*_2_ highlights the fractal dimensionality of the attractor and demonstrates the strangeness of the complex geometrical structure^34^. We note that this *D*_2_ value is already higher than that of several canonical chaos structures such as the Hénon map (at 1.21), the logistic map (at 0.5), and the Kaplan-Yorke map (at 1.42), and is even close to that of Lorenz chaos (at 2.05)^36^.

Furthermore the waveform unpredictability can be characterized by the second-order Renyi approximation of the Kolmogorov entropy *K*_2_:


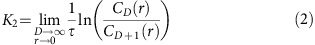


where *τ* is the time series sampling rate, a measurement of the system uncertainty and a sufficient condition for chaos^38^. A positive *K*_2_ is characteristic of a chaotic system, while a completely ordered system and a totally random system will have *K*_2_=0 and *K*_2_=∞ respectively. With the Grassberger-Procaccia algorithm, *K*_2_ is calculated as ≈0.17 ns^−1^ or expressed equivalently as ≈1.52*τ*_omo_^−1^, representing that the mean divergence rate of the orbit section (with adjoining point pairs in the phase space) is rapid within 1.52 times the fundamental OMO period. It characterizes the gross expansion of the original adjacent states on the attractor^38^ and, therefore, indicates the significant unpredictability in the dynamical process of such solid-state systems.

### Theoretical simulation of chaos

To further support the physical observations, we model the dynamics of the optomechanical photonic crystal cavity system under the time-domain nonlinear coupled mode formalism, taking into account the OMO^21^, TPA^31^, free-carrier and thermo-optic dynamics^30^,^31^:


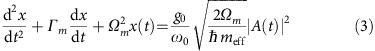



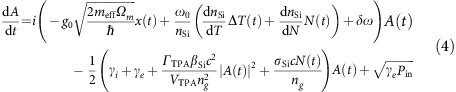



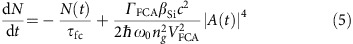



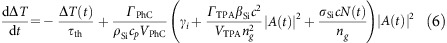


where *x*, *A, N* and Δ*T* represent respectively the motional displacement, the intracavity **E**-field amplitude, the free-carrier density and the cavity temperature variation. *δω*=*ω*_L_−*ω*_0_ is the detuning between injection light, *ω*_L,_ and photonic crystal cavity resonance, *ω*_0_, and *P*_in_ is the injected optical power (detailed in Supplementary Note 3, Supplementary Table 1). Equation (3) describes the optically driven damped mechanical harmonic oscillation with self-sustained OMO oscillations when pumped above threshold. The mechanical oscillations then in turn result in modulation of the intracavity optical field (first term on the right-hand side of equation (4)). On the other hand, the plasma induced thermal-optic effect and free-carrier dispersion in the cavity (second and third terms on the right-hand side of equation (4)) lead to another amplitude modulation of the intracavity field. Here, the high-density Drude plasma is generated by the strong TPA in silicon (equation (5)). With the increased intracavity power, the free-carrier dispersion effect leads to blue-shifts of the cavity resonance while the free-carrier absorption induced thermo-optic effect results in red-shifts of the cavity resonance. The dynamical interplay between these two effects results in the regenerative SOM (refs 30, 31). The mechanism is detailed in Supplementary Fig. 6 and Supplementary Note 6. We note that our photonic crystal design ensures that the characteristic timescales of the SOM and OMO oscillations are on the same order of magnitude (Supplementary Fig. 8), strengthening the effective inter-oscillator coupling. The coexistence of OMO and SOM mechanisms adds extra degrees of freedom to the dynamic space of system and results in increased susceptibility to destabilization (detailed in Supplementary Note 2)^16^,^18^,^21^.When the drive power is between the SOM and OMO thresholds, TPA-associated amplitude modulations disrupt the OMO rhythm, breaking the closed OMO limit cycles and creating the non-repeating chaotic oscillations. On the other hand, if the frequency ratio between OMO and SOM is close to a rational value, they will lock each other based on the harmonic frequency locking phenomena^39^,^40^. Consequently, different sub-harmonic *f*_omo_ states are also observed in Fig. 3. Effects of the Drude free-carrier plasma, the detuning *δω*, the optomechanical coupling strength *g*_0_ and the injected drive power *P*_in_ on the chaotic transitions and routes are detailed in Supplementary Notes 4–7.

Figure 4e shows the dynamical distribution map simulated numerically and parametrically with the normalized detuning *δω*/*γ*_i_ versus injection power *P*_in_, where *γ*_i_ is the intrinsic cavity linewidth from linear losses. The various regimes are denoted with different colours, and rigorously identified through entropic analysis of the temporal waveform uncertainty and periodicity of the Fourier spectrum. The temporal waveforms are often strongly periodic in the limit cycle states (such as OMO and USP) and have low entropy (indicated by the darker colours), while the chaotic oscillation has a significant uncertainty and high entropy (indicated by the brighter colours). In Fig. 4e, the crescent-shaped region (in bright orange) indicates the parametric conditions of the complex chaos state. Around this region, there are rich transitional dynamics related to chaos, thereby enabling different routes into or out of chaos with different parameter scanning approaches. When the pump power is 1.26 mW, the numerical model predicts a bifurcation transition to chaos via states OMO-USP-*f*_omo_/3-*f*_omo_/2-chaos-SOM as a function of detuning, in a qualitative agreement with the experimental observations. It is of note that the system of coupled equations does not involve any initial noise terms, illustrating the deterministic nature of the obtained chaotic solutions.

## Discussion

We demonstrate chaos generation in mesoscopic silicon optomechanics achieved through single-cavity coupled oscillations between radiation-pressure- and two-photon-induced free-carrier dynamics. Chaos generation is observed at 60 fJ intracavity energies, with a correlation dimension *D*_2_ determined at ∼1.67. The maximal LE rate is measured at 2.94 times the fundamental OMO, and the second-order Renyi estimate of the Kolmogorov entropy *K*_2_ is determined at 1.9 times the fundamental OMO, both showing fast adjacent trajectory divergence into the chaotic states. Furthermore, we route the chaos through unstable states and fractional subharmonics, tuned deterministically through the drive-laser detuning and intracavity energies. These observations set the path towards synchronized mesoscopic chaos generators for science of nonlinear dynamics and potential applications in secure and sensing application, in light of recent works about gigahertz OMOs^41^ and synchronization of coupled optomechanical oscillators^42^.

## Methods

### Device design and fabrication

The optomechanical photonic crystal cavity is fabricated with a CMOS-compatible process on 8-inch silicon wafers at the foundry, using 248 nm deep-ultraviolet lithography and reactive ion etching on 250 nm thickness silicon-on-insulator wafers. To realize the critical 120 nm slot width, the resist profile is patterned with a 185 nm slot linewidth, then transferred into a sloped oxide etch. The resulting bottom 120 nm oxide gap is etched into the silicon device layer through tight process control. Multiple planarization steps enable high-yield of the multi-step optomechanical photonic crystal fabrication. The optical input/output couplers are realized with silicon inverse tapers and oxide overcladding coupler waveguides. The optomechanical photonic crystal cavities are released by timed buffered oxide etch of the undercladding oxide.

### Measurement set-up

The drive laser is a tunable Santec TSL-510C laser (1,510–1,630 nm), which is also used to measure the optical transmission spectra. The drive laser is first amplified by a C-band erbium-doped fibre amplifier and then injected into the slot-type photonic crystal cavity with a coupling lens placed on an adjustable 25-nm precision stage. A—fibre polarization controller with a prism polarizer selects the transverse-electric polarization state for the cavity mode. The output transmission of the photonic crystal cavity is collected into fibre through a coupling lens, an optical isolator, and then into a New Focus (Model 1811) detector, before an electronic spectrum analyzer (Agilent N9000A) and time-domain digital oscilloscope (Tektronix TDS 7404) characterization and statistical analysis.

### Numerical simulations

The coupled equations (1)–(4), 2, 3, 4 are numerically solved with the fourth-order Runge-Kutta algorithm. The time discretization is set as 10 ps and each simulated temporal waveform contains 10^7^ data points (100 μs). The simulated RF spectrum is calculated with the fast Fourier transform method, which is a discrete Fourier transform algorithm to rapidly convert a signal from its time domain to a representation in the frequency domain. In frequency domain, we can easily get the spectral characteristics of the signal. The long time span of the temporal waveform (at 100 μs) is also necessary for resolving the 25 kHz spectral features and converging in the subsequent statistical analyses.

### Data availability

The authors declare that the data supporting the findings of this study are available within the paper and its Supplementary Information files.

## Additional information

**How to cite this article:** Wu, J. *et al*. Mesoscopic chaos mediated by Drude electron-hole plasma in silicon optomechanical oscillators. *Nat. Commun.* **8**, 15570 doi: 10.1038/ncomms15570 (2017).

**Publisher's note**: Springer Nature remains neutral with regard to jurisdictional claims in published maps and institutional affiliations.

## Supplementary Material

Supplementary Information Supplementary Notes, Supplementary Figures, Supplementary Table and Supplementary References
