# Supplementary material for: Mesoscopic chaos mediated by Drude electron-hole plasma in silicon optomechanical oscillators
Source: Nat Commun. 2017 Jun 9;8:15570. doi: 10.1038/ncomms15570 (PMC5477489; doi:10.1038/ncomms15570)
Supplement: Supplementary Information — Supplementary Notes, Supplementary Figures, Supplementary Table and Supplementary References [file ncomms15570-s1.pdf]

Supplementary Note 1: Experimental data and chaos identification

In this section we analyze the experimentally recorded temporal waveforms. There are various kinds of noise processes in the experimental setup and measurements which can affect the process of chaos identification. Therefore it is necessary to reduce the noise in temporal waveforms. Here, the length of temporal waveforms is recorded over 10^5 data points with a high-sensitivity photodiode and a digital oscilloscope at 10 Gs/s sampling rate. We use the state-space averaging method to discriminate the stochastic noise from the chaotic waveform [1,2]. In this method, the *n*th data point of the recorded temporal waveforms is denoted by X_n. The noise-reduced data points are expressed as $\tilde{X}_n$. A sliding window is tied to X_n with a width ±*m* points in the state-space. Then, $\tilde{X}_n$ is expressed as [S1,S2]:

$$\tilde{X}_n = \frac{\sum_{k=m}^{N-m} X_k \omega_n(k)}{\sum_{k=m}^{N-m} \omega_n(k)}, \text{ and } \omega_n(k) = \exp(-\sum_{j=-m}^{m} (X_{k-j} - X_{n-j})^2 / \sigma_{noise}^2) \qquad (1)$$

The data point $\tilde{X}_n$ averages the dataset X_n in a sliding ±*m* points window around X_n in the *m*-dimensional embedding space. $\omega_n(k)$ gives the nearby points different weight; the nearer points have more weight. For our data, *m* is set as 10, σ_{noise} is the noise standard deviation and at 0.2 optimally, while maintaining the dynamical complexity of chaotic temporal waveform as shown in Figure 2b of the main text.

To identify the chaotic dynamics, we calculated the Lyapunov exponents (LEs) of the recorded time series. The LEs are critical measures of dynamical stability of a system. In mathematics, the LEs characterize quantitatively the rate of separation of infinitesimally close trajectories. Negative LEs are characteristic of dissipative stable systems. The more negative the LEs, the better the stability. Zero LE is characteristic of a conserved stable system. Positive LEs reveal the orbit is unstable and chaotic. Nearby points, no matter how close, will diverge to any arbitrary separation. Two trajectories in phase space with initial separation δZ_0 diverge (being treated with the linear approximation) at a rate given by [2]: $|\delta Z(t)| \approx e^{\lambda t} |\delta Z_0|$, where λ is the LE. In the multi-dimensional phase space, the rate of separation should be different for different orientations of the initial separation vector. Thus

there is a spectrum of Lyapunov exponents [3]. The largest of the LEs can be called the maximal Lyapunov exponent (MLE), and determines the notion of predictability for a dynamical system. A positive MLE is usually taken as an indication that the system is chaotic [2].

We next implement the canonical Grassberger-Procaccia (G-P) algorithm [4-6] to estimate the correlation dimension D_2 (close to the fractal dimension of attractor) and the K_2 entropy (close to the Kolmogorov entropy) [4-6]. For example, if we have a set of random points being distributed on a triangle face embedded in three-dimensional space or four-dimensional space, the correlation dimension will always be 2. The correlation dimension has the advantage of quick calculation, of only needing a small number of points, and often agreeing well with the results of other dimension estimation methods [6]. The convergence of the G-P algorithm is sufficient to demonstrate chaos and estimate its finite correlation dimension. During calculation, the value of D_2 could be calculated from the integral $C_D(r)$:

$$C_D(r) = \frac{1}{N^2}\left(\sum_{n,m}(d(X_n, X_m) < r)\right) \tag{2}$$

This formula is a numerical computation of the average number of vectors that could be found within a sphere of radius r around a given vector. Distance d is the Euclidian norm. From the analysis of Grassberger-Procaccia [4-6], we have:

$$D_2 = \lim_{\substack{D\to\infty \\ r\to 0}} \frac{d\ln(C_D(r))}{d\ln(r)} \quad \text{and} \quad K_2 = \lim_{\substack{D\to\infty \\ r\to 0}} \frac{1}{\tau}\ln\left(\frac{C_D(r)}{C_{D+1}(r)}\right) \tag{3}$$

where τ is the sampling rate of the time series. The formulas converge with increasing D size. In our calculation we show the results for D between 15 and 20.

Supplementary Note 2: Detailed route into and out of chaos

The governing dynamical equations have been described in Ref. [7-9]. In our case, the small modal volume and high Q of photonic crystal (PhC) cavity result in the high intensity of local optical field, optomechanical oscillations [8] and significant two-photon absorption (TPA) effect [7]. Next, TPA mainly produces the heat and free carriers [7, 9]. The dispersion induced by the Kerr effect can be ignored since it is orders of magnitude weaker than the above nonlinearites in silicon [7, 8]. Next, to obtain the main text equations (3) to (6), we

consider the mechanical system is a second-order system (represented by x). With the slowly-varying envelope approximation, the PhC intracavity field is represented by A and the free-carrier density (represented by N) and temperature variation (represented by ΔT) of the PhC cavity is added. One then adds two equations into the equations given in Ref. [7-9], which later become the time-domain nonlinear coupled equations (3) to (6) shown in the main text.

To further illustrate the detailed route into and out of chaos [10-27], a typical sequence of dynamics is given in Supplementary Figure 1. From top to bottom, the injection wavelength detuning is (**a**) 0.910 nm, (**b**) 1.270 nm, (**c**) 1.394 nm, (**d**) 1.831 nm, (**e**) 2.071 nm, (**f**) 2.285 nm, (**g**) 2.406 nm, (**h**) 4.070 nm and (**i**) 5.910 nm respectively. The system is measured both in the frequency-domain (the radio frequency (RF) spectra and in the time-domain (the temporal waveforms and the phase portraits). Based on the unique frequency-temporal characteristics, the dynamical states can be identified. In Supplementary Figure 1, several dynamical states can be identified as: (**a**) the optomechanic oscillation (OMO) state, (**b**) the unstable pulsation state (USP), (**c**) the $f_{omo}/4$ state, (**d**) the $f_{omo}/3$ state, (**e**) the $f_{omo}/2$ state, (**f**) the chaos state (chaos), (**g**) the $f_{omo}/2$ state again, (**h**) the self-induced optical modulation (SOM) state, and (**i**) the stable state (S) respectively. Specifically, for the OMO state, as shown in Supplementary Figure 1a, there is a weak but clear frequency peak shows out at 112MHz. For USP state as shown in Supplementary Figure 1b, the pulses are unstable in both amplitude and period. This is the reason why it is referred to unstable pulses (USP) state. Comparing Supplementary Figure 1b and Supplementary Figure 1c, one can find a resemblance of the temporal waveforms and the phase portraits between the USPs state and $f_{omo}/4$ state. It indicates that the USP state is a transition state into the $f_{omo}/4$ state in PhC-OM system. In brief, the OMO cavity shows rich nonlinear dynamics, following a route of OMO - USP - $f_{omo}/4$ - $f_{omo}/3$ - $f_{omo}/2$ – chaos - $f_{omo}/2$ – SOM - S. Three 2D RF spectral evolution maps of nonlinear and chaotic dynamics, measured with different injection powers, are shown in Supplementary Figure 2(a, b & c). Based on these evolution maps, one can see that the parameter range covered by the various nonlinear dynamical states is gradually increased and extended to the longer wavelength region with increasing the injection power. Furthermore,

we also demonstrate another dynamical route of an optomechanical photonic crystal cavity by scanning the injection power and with the initial low-power detuning Δ fixed at 0.87 nm. In Supplementary Figure 3, from top to bottom, the measured dynamical states follow the sequences of: (**a**) the $f_{omo}/3$ state, (**b**) the chaos state, (**c**) the $f_{\mathrm{omo}}/2$ state, (**d**) the SOM state, and finally (**e**) the stable state (pre-OMO state without dynamical fluctuations) S.

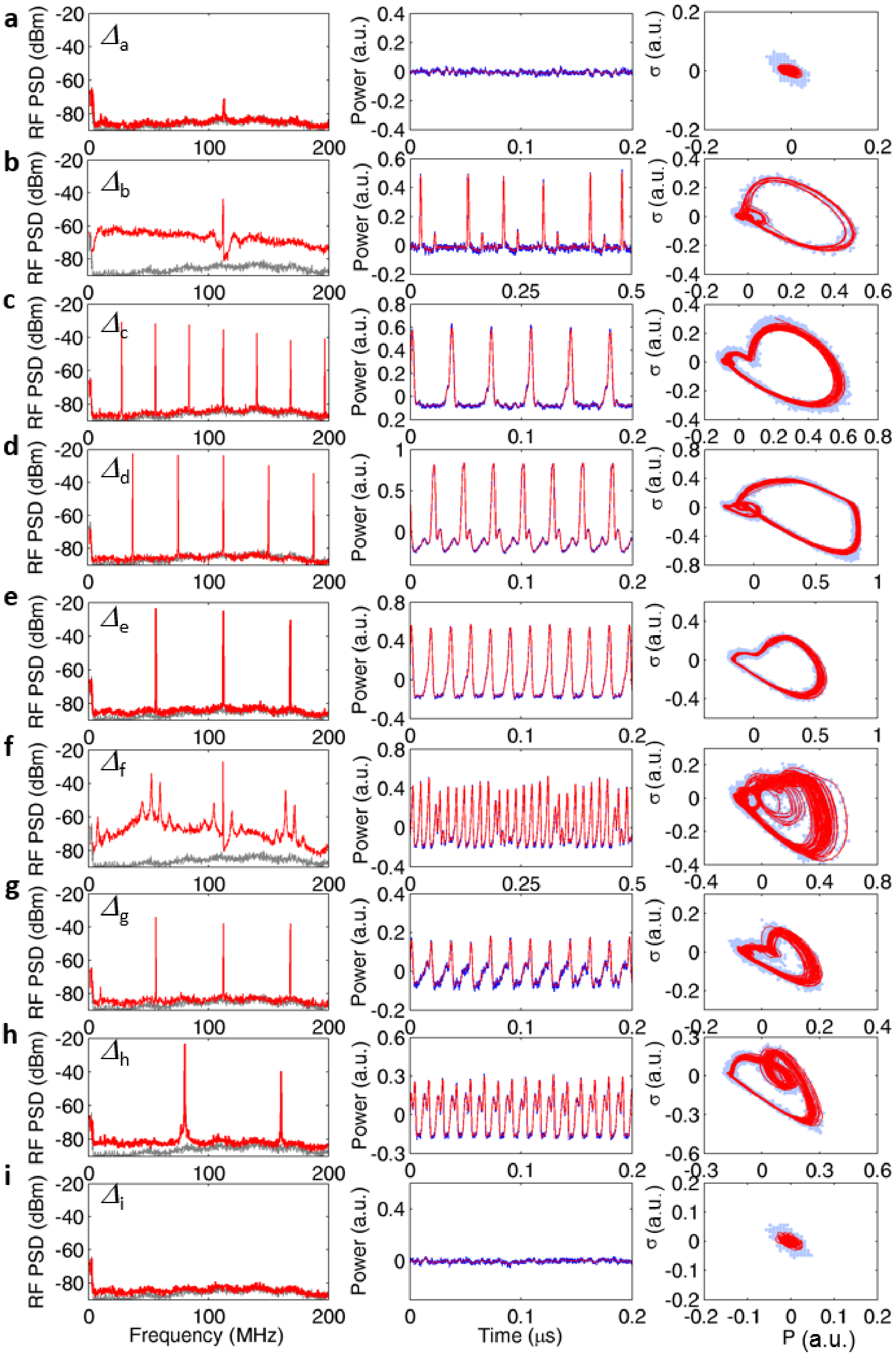

Supplementary Figure 1. **Detailed dynamical transition.** The detuning Δ varies from (**a**) to (**i**) as: (**a**) 0.910 nm, (**b**) 1.270 nm, (**c**) 1.394 nm, (**d**) 1.831 nm, (**e**) 2.071 nm, (**f**) 2.285 nm, (**g**) 2.406 nm, (**h**) 4.070 nm, and (**i**) 5.910 nm respectively. The injection power is kept constant

at 1.26 mW under different injection detuning. The first column shows the measured radio frequency (RF) spectra of different dynamical states, where the grey curves are the background noise floor. The second and third columns show the corresponding temporal waveforms and phase portraits, where the blue dots are the measured data and the solid red curves are the noise-reduced trajectories.

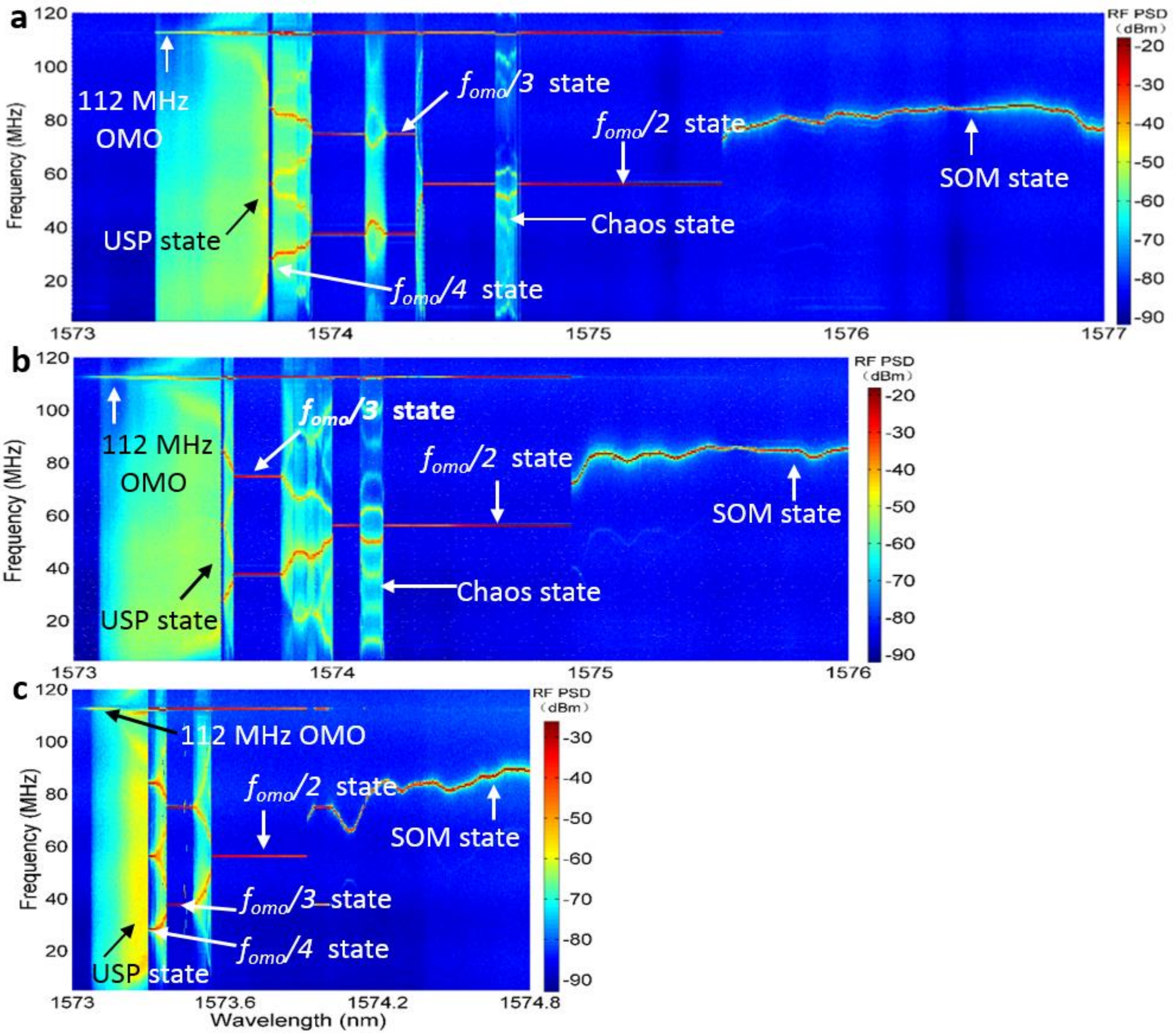

Supplementary Figure 2. **2D RF spectra evolution map of nonlinear dynamics.** The injection power decreases from top to bottom as: (**a**) 0.8 mW, (**b**) 0.5mW, and (**c**) 0.32 mW respectively. The dynamics are detailed as optomechanical oscillation (OMO) state, unstable pulse (USP) state, $f_{omo}/4$ state - $f_{omo}/3$ state, chaos state, $f_{omo}/2$ state and self-induced optical modulation (SOM) state, respectively.

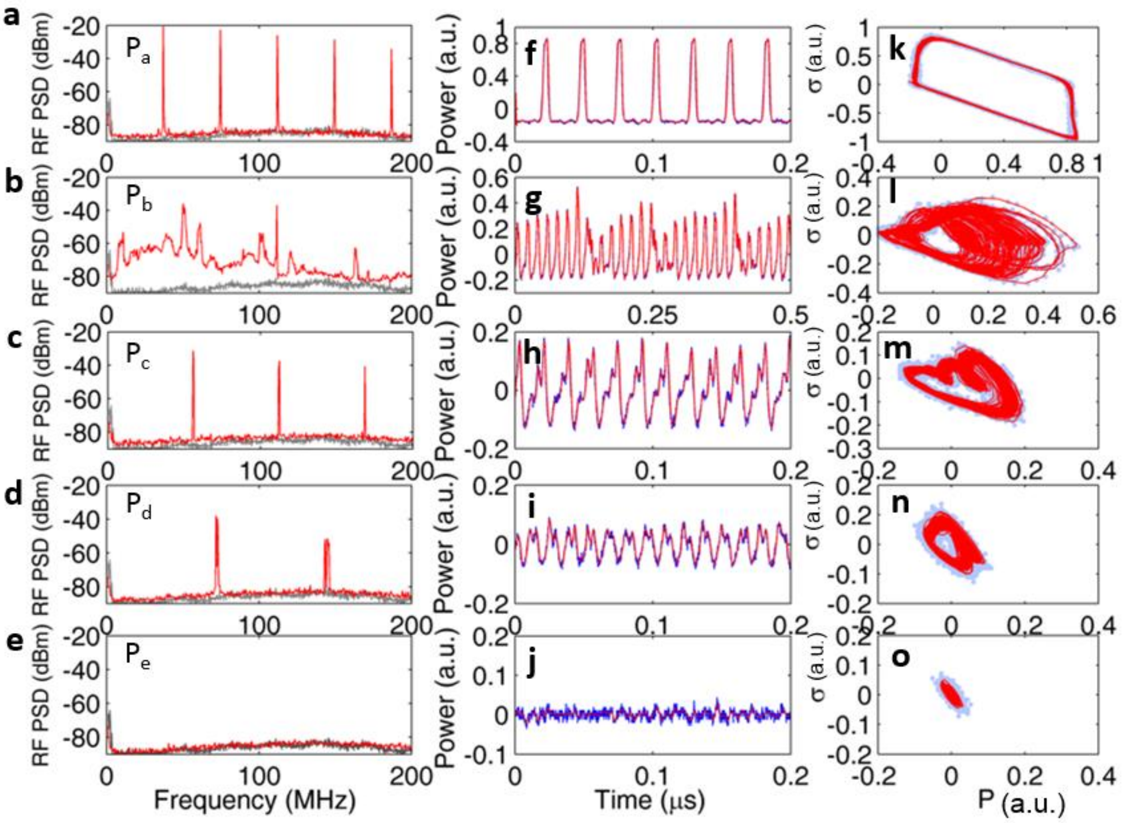

Supplementary Figure 3. **Dynamical evolution under different injection powers.** The injection power increases from top to bottom as: (**a**) 0.68 mW, (**b**) 0.43 mW, (**c**) 0.38 mW, (**d**) 0.22 mW and (**e**) 0.15 mW respectively. The initial low-power detuning Δ is set at 0.87 nm. The (**a**, **b**, **c**, **d** and **e**) shows the measured RF spectra, where the grey curves are the background noise floor. The (**f**, **g**, **h**, **i** and **j**) show the noise-reduced temporal waveforms (red curves), and the (**k**, **l**, **m**, **n** and **o**) show the corresponding phase portraits.

Physically, there are two mechanisms in our slot-type optomechanical PhC chip which consists of the micro-mechanical resonator and the high *Q/V* PhC optical resonator (the detailed coupling scheme is shown in Supplementary Figure 4). Firstly, for a micro-mechanical resonator, with high *Q/V* ratio and sub-wavelength optical confinement, large intracavity radiation pressure forces can modify the motion of micro-mechanical resonators (labeled as $\boldsymbol{x}$ in equation 3 of the main text) [28, 29]. When the input optical power exceeds the intrinsic mechanical damping losses, a self-sustained oscillation can be formed [29, 30], and is called the OMO limit-cycle from the dynamical point of view. The OMO limit-cycle modulates the PhC optical field (labeled as $\boldsymbol{A}$ in equation 4 of the main text), and is read out by measuring the optical transmission signal.

Secondly, the high *Q/V* PhC optical resonator is also affected by a series of silicon-based nonlinearities (the nonlinear coupling is shown in Supplementary Figure 4), such as the two-photon absorption (TPA), Drude plasma free-carrier dispersion (FCD), free-carrier absorption (FCA), and the thermo-optic effect [2]. Mainly, the PhC optical mode resonance can be blue-shifted by FCD and red-shifted by the TPA- and FCA-induced thermo-optic effect [7, 8]. This generates a competing relationship between nonlinearities, resulting in a temporal modulation on the PhC optical field. This modulation is called the self-induced optical modulation (SOM) [7, 8]. Next, let us illustrate the SOM process in detail. Initially, a slight red detuning exists between PhC cavity and input laser frequency. A large TPA effect is introduced by the strong optical field in the PhC cavity. TPA generates a large free carrier density (labelled as $\boldsymbol{N}$ in equation 5 of main text). Then, the FCD mechanism and the FCA mechanism will dissipate the free carriers. Firstly, FCD mechanism will cause a rapidly blue-shift of PhC resonance. Secondly, the TPA and FCA will also heats the PhC cavity (labeled as ΔT in equation 6 of the main text), and introduce a slow red-shift of the PhC resonance through the thermo-optic effect. This red-shift will eventually stop the rapid blue-shift, leading to a red-shift of the PhC resonance. Thirdly, eventually the PhC resonance red-shifts over the input laser frequency, and results in a rapid drop of PhC intracavity optical field with a large residual red-shift. Fourthly, the large residual red-shift and thermal energy slowly decays through thermal radiation and thermal conduction of in the silicon structure. Finally, the PhC cavity has cooled and is slightly red-detuned again from the input laser frequency. A SOM type limit-cycle consequently forms. Such SOM limit-cycle will also modulate the PhC optical field. From the dynamical point of view, the OMO limit-cycle and SOM limit-cycle are the degrees of freedom of the PhC optical field. If OMO is absent, the PhC chip will output the periodic SOM signal as shown in Ref. [7, 8]. The coexistence of OMO limit-cycle and SOM limit-cycle adds extra degrees of freedom to the dynamical space of the system and easily destabilizes the dynamics [19, 20, 22]. With effective coupling between OMO and silicon nonlinearities, and enough drive power above the OMO and SOM thresholds, TPA-associated modulations disrupt the OMO rhythm, breaking the OMO limit cycles and creating the non-periodical chaotic oscillations.

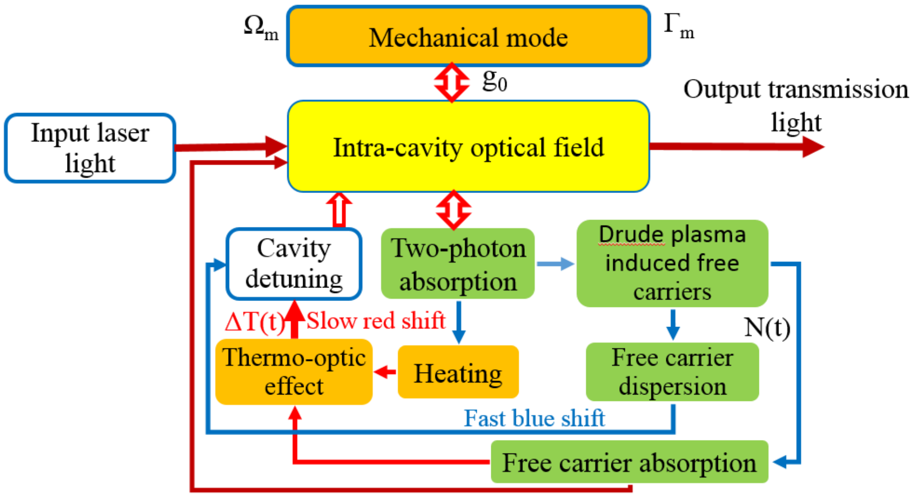

Supplementary Figure 4. **The coupling mechanism of various nonlinearities.** The nonlinearities and mechanical mode of the photonic-crystal optomechanical chip are all considered in our theoretical model [equations (3) to (6) of the main text], where the g_0 is the optomechanics coupling strength, Γ_m is the mechanical dissipation rate, Ω_m is the mechanical angular frequency, ΔT is the cavity temperature variation and N(t) is the free-carrier density. The intracavity optical field is modulated simultaneously both by the mechanical oscillation and by various silicon nonlinearities, such as the two-photon absorption, Drude plasma free-carrier dispersion, free-carrier absorption and the thermo-optic effect. Competitive coupling relationship exists between these nonlinearities, resulting in the complex chaos dynamics.

Supplementary Note 3: Parameters of theoretical modelled and modeled transitional dynamical states

Table 1 summarizes the parameters used in the numerical simulation under the nonlinear coupled mode theory formalism [31, 32]. Material constants are taken from Refs. [7, 8, 33, 34]; other parameters are obtained by measurements, finite-element method (FEM) simulation (COMSOL Multiphysics), and the combination of simulations and experimental fitting. Note that the Kerr nonlinearity and Raman scattering are much weaker than the other nonlinearities and hence they are not included in our model [7, 8, 33, 34].

Supplementary Table 1. **Modelling parameters.**

Parameter	Physical meaning	Value	Source

β_{Si}	Two-photon absorption (TPA) coefficient	8.4×10^{-12} m/W	material constant
n_g	Group index	3.476	material constant
σ_{Si}	Free-carriers absorption (FCA) cross section	1×10^{-21} m^2	material constant
n_{Si}	Refractive index of Si	3.476	material constant
ρ_{Si}	Density of material Si	2.33×10^{3} kg/m^2	material constant
c_p	Specific heat capacity	700 J/(kg K)	material constant
$\frac{dn_{Si}}{dT}$	Thermo-optic coefficient	1.86×10^{-4} K^{-1}	material constant
$\frac{dn_{Si}}{dN}$	Free carrier coefficient	-1.73×10^{-27} m^3	material constant
λ_o	Resonance wavelength	1572.8 nm	measured
γ_i	Linear loss (radiation & abs.)	19 GHz	measured
$\Omega_m/2\pi$	Mechanical frequency	112 MHz	measured
g_0	Vacuum optomechanics (OM)coupling strength	690 kHz	estimated
Γ_{TPA}	TPA confinement factor	0.8012	FEM
V_{TPA}	TPA mode volume	6.4×10^{-19} m^3	FEM
m_{eff}	Mechanical osc. effective mass	2.4×10^{-14} kg	FEM
Γ_{FCA}	FCA confinement factor	0.79	FEM
V_{FCA}	FCA mode volume	6.9×10^{-19} m^3	FEM
Γ_{PhC}	Thermal confinement factor	0.769	FEM
τ_{fc}	Free-carrier lifetime	150 ps	fitted
τ_{th}	Thermal dissipation life time	9.7 ns	fitted
$\Gamma_m/2\pi$	Mechanical dissipation rate	110 kHz	estimated

$\gamma_e/2\pi$	External coupling rate	2.2 GHz	estimated
V_{PhC}	Thermal mode volume	1×10^{-18} m^3	estimated

Supplementary Figure 5 shows an example of the simulated chaos and the associated dynamical transitions of the two-photon optomechanical photonic crystal cavity. Supplementary Figure 5a shows the pure OMO state. Next, Supplementary Figure 5b, 5c, 5d show the $f_{\text{omo}}/2$ state, $f_{\text{omo}}/3$ state and $f_{\text{omo}}/4$ state respectively. Clear sub-harmonic frequency peaks can be observed in these Fourier transformed power spectra, and the corresponding phase portraits also characterize clearly the limit-cycle features. Supplementary Figure 5e gives the simulated chaotic oscillation; its power spectrum distributes continuously, being quite different from the discrete distribution spectrum. The temporal waveform (Supplementary Figure 5j) behaves intricately. Moreover, the corresponding phase orbit (Supplementary Figure 5o) consistently winds and stretches in the basin of the strange attractor; this induces the fractal structure [2, 6, 35]. These simulations confirm qualitatively the experimental observations of Figure 2 in the main text. In addition, we emphasize that the obtained chaos is deterministic since the simulation equations (3)-(6) are deterministic and do not contain any noise terms.

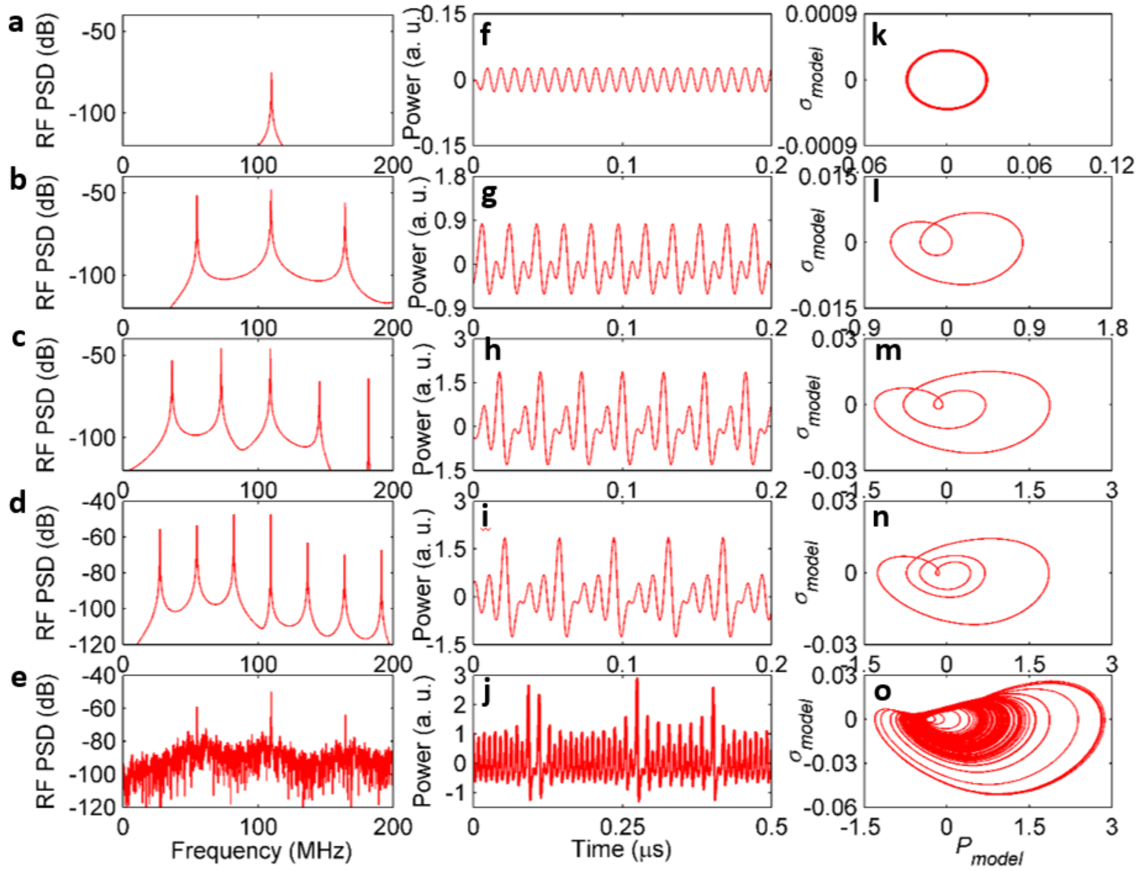

Supplementary Figure 5. **Numerical simulations of chaotic output.** (**a-e**): Fourier transformed power spectrum. (**f-j**): temporal waveform. (**k-o**): corresponding phase portrait, where the injection power P_{in} is 1 mW and normalized detuning $\delta\omega/\gamma_{\mathrm{i}}$ varies from top to bottom as: (**a, f, k**) 2, (**b, g, i**) 5.5, (**c, h, m**) 8.65, (**d, i, n**) 7.95, and (**e, j, o**) 10. The mechanical frequency $\Omega_{\mathrm{m}}/2\pi$ value is 110 MHz and other parameters are given in Supplementary Table 1.

Supplementary Note 4: Effects of the optomechanics coupling strength g_0 in chaotic transition and routes

The large optomechanical (OM) coupling strength (g_0) means the strong coupling between optical field and mechanical motion in the PhC nanocavity. The localized mode of the PhC-OM cavity has a much larger g_0 than regular large Fabry-Perot cavities. The OM coupling strength (g_0) is estimated using the phase modulation method described in [36, 37]:

$$g_0^2 \approx \frac{1}{2\bar{n}_{th}} \frac{\Phi_0^2 \Omega_{mod}^2}{2} \frac{S(\Omega_m) \times \Gamma_m/4}{S(\Omega_{mod}) \times RBW} \qquad (4)$$

where $\bar{n}$ is the average phonon occupancy, ϕ_0 is the phase modulation amplitude, and Ω_{mod} is

the modulation angular frequency. The proportion between $S(\Omega_m)$ and $S(\Omega_{mod})$ is obtained by measuring the peak spectral power of the mechanical oscillation and that of phase modulation signals. RBW is the resolution bandwidth of the spectrum analyzer, and $\Gamma_m/2\pi$ is the dissipation rate of the mechanical oscillator. For the chip used in this manuscript, the value of g_0 is determined to be about 690 kHz.

To further illustrate the effect of the coupling strength g_o on the chaos generation, we examine theoretically a varied coupling strength g_o between the OMO and photonic crystal cavity nonlinearities based on the numerical model. The coupling between resonators usually leads to the generation of complex nonlinear dynamics [38-40]. Supplementary Figure 6 shows the simulated dynamical evolution under different g_o values. First, the system has pure OMO transmission signal for a small coupling strength g_o. Next, when g_o grows over a specific value (about 330 kHz), discrete frequency components can be observed at the locations of multiples of $f_{omo}/2$ or $f_{omo}/4$. It means the system has evolved into the $f_{omo}/2$ or $f_{omo}/4$ states. Along with the increase of g_o value, the OMO couples strongly with cavity nonlinearities. When g_0 grows over a threshold value (about 670 kHz), the system shows complex evolution and finally transits into the chaos state. Thus, sufficiently strong g_o is a necessary condition for the chaos generation. In addition, it should be noted the threshold g_o value is dependent on the varied injection conditions, such as the injection power and detuning. A theoretical analysis has been addressed for the physical reasons of this deviation from eigenmode mechanical oscillations [41]. This is the reason why the chaos state only appears at specific ranges of injection detunings and powers as shown experimentally in Figure 4, Supplementary Figures 1 & 3.

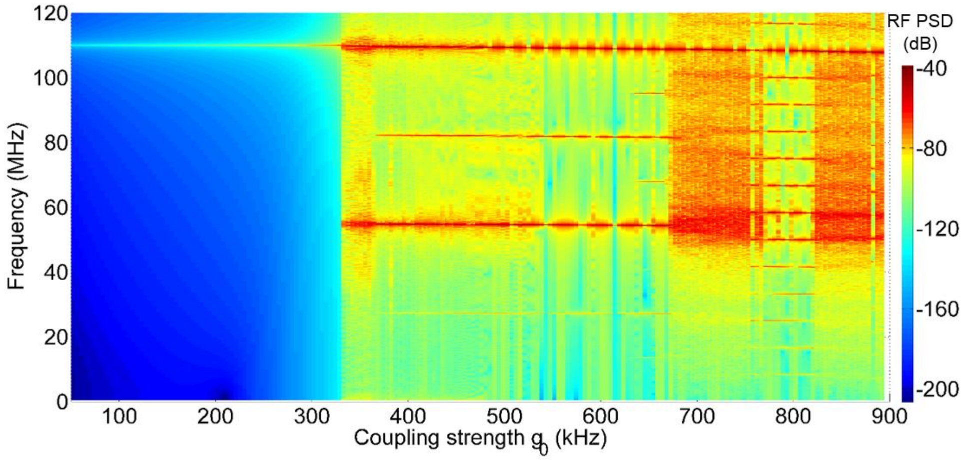

Supplementary Figure 6. **Investigation of dynamics evolution under different value of g_0.** Simulated 2D radio frequency (RF) spectral map with varied dynamics for different coupling strength g_0 values, with the injection power fixed at P_{in} = 1 mW and the normalized detuning at 10. The mechanical frequency $\Omega_m/2\pi$ value is 110 MHz and the other parameters are given in Supplementary Table 1. The inset shows the fine evolution of the RF frequency around 110 MHz, with the slight RF frequency shift from increased g_0 and stronger optical gradient force.

Supplementary Note 5: Effects of the laser-cavity detuning in chaotic transition and routes

Supplementary Figure 7 illustrates the simulated 2D RF spectral map with dynamics evolution under different detunings $\delta\omega/\gamma_i$, with the injection power P_{in} at 1.26 mW. In the Supplementary Figure 7, the left regime is the OMO state in the range of $\delta\omega/\gamma_i$ less than 3.5. The pure OMO signal is present at 112 MHz. Second, in the range of 3.5 < $\delta\omega/\gamma_i$ < 8.5, the USP state appears. The identification of the USP state could be carried out by combining the spectral feature and the temporal characteristics together. The spectral distribution of the USP state is continuous, but the temporal waveform of the USP state is irregular pulsing, as shown in Supplementary Figure 1b. Furthermore, complex high-order harmonics are also interspersed among the USP region. Third, the $f_{omo}/3$ state appears in a small window at $\delta\omega/\gamma_i \approx 8.5$. In addition, the $f_{omo}/2$ state appears in the range of $\delta\omega/\gamma_i \approx 10$. Significant $f_{omo}/2$ components appear in the RF spectra but have associated relatively weak $f_{omo}/4$ components. Next, the system evolves into the chaos state in the range $\delta\omega/\gamma_i \approx 11$. As shown by the RF

spectra, the chaos evolution is dominated by both the OMO and the SOM together, indicating the chaos comes from the coupling between the OMO and SOM oscillation. Finally, for $\delta\omega/\gamma_i$ greater than 12, the SOM state becomes the dominant dynamics while the OMO oscillation disappears. Overall, the above numerical results predict a dynamical transition to chaos of OMO - USP - $f_{omo}/3$ - $f_{omo}/2$ - chaos - SOM as a function of detuning. This modeled dynamics evolution is in qualitative agreement with the experimental observations in Figure 1d.

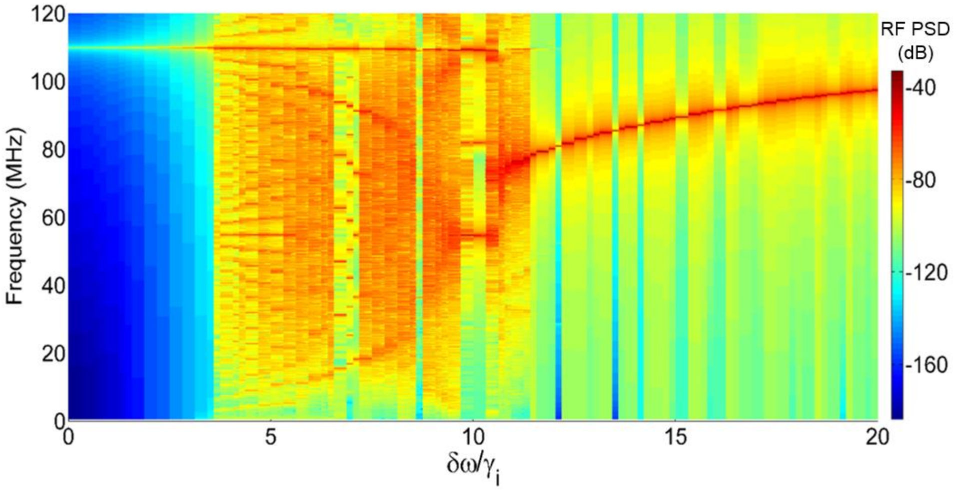

Supplementary Figure 7. **The dynamics evolution under different detunings.** Simulated 2D radio frequency (RF) spectral map with various dynamics under normalized laser-cavity detunings from 0 to 20, with the injection power P_{in} = 1.26 mW and optomechanical coupling strength g_0 = 690 kHz. The other parameters are shown in Supplementary Table 1.

Supplementary Note 6: Co-located two-photon-induced Drude free-carrier plasma and optomechanical dynamics

To investigate the coupling between the OMO and silicon nonlinearities, we separate artificially the OMO and silicon nonlinearities [8, 28-30, 36, 42] in our numerical simulations, and then recombine them together again. First, we get a simplified OM oscillator by setting all photonic crystal cavity nonlinear coefficients to zero, as Γ_{TPA} = 0, Γ_{FCA} = 0, Γ_{phc} = 0, dn_{Si}/dT = 0 and dn_{Si}/dN = 0, respectively. Supplementary Figure 8a shows the corresponding OMO evolution under different injection powers and the normalized detuning fixed at 10. It shows that no complex nonlinear dynamics are observed, and the pure OMO is present at the intrinsic 110 MHz frequency and with a monotonic increase in RF power with increasing

injection power. Second, by setting g_o at 0 (without OMO), we numerically obtain a purely photonic crystal cavity regenerative modulation. Supplementary Figure 8b shows the cavity dynamical evolution under different injection powers. It can be seen that there is no observable self-induced optical modulation (SOM) [7, 8] in the relatively low P_{in} condition for $P_{\mathrm{in}} < 1.56$ mW. For P_{in} larger than 1.56 mW, the SOM and its harmonics emerge at the frequency 66 MHz and 132 MHz, and gradually decrease along with the increase of P_{in} due to the larger thermal effects and longer relaxation time to the periodic origin state. It should be noted that the 66 MHz SOM is comparable with the half of OMO frequency $f_{\mathrm{omo}}/2$ at 55 MHz, supporting the occurrence of the $f_{\mathrm{omo}}/2$ state.

Third, Supplementary Figure 8c shows the dynamical evolution of the optomechanical cavity system when combining the OMO and the photonic crystal Drude electron-hole plasma nonlinearities together. The chaos state is present at the broad area of the medium P_{in} condition (labelled region *II* for $1.56 < P_{\mathrm{in}} < 2.18$ mW), originating from the large coupling between OMO and SOM. For higher P_{in} condition (labelled region *III* for $P_{\mathrm{in}} > 2.18$ mW), discrete frequency components are present at multiples of $f_{\mathrm{omo}}/2$, demonstrating the $f_{\mathrm{omo}}/2$ state. Generally, in a system that has two intrinsic frequencies, harmonic frequency locking will occur when the frequency ratio is close to a rational value [43, 44]. For example, in our PhC-OM chip, the frequency SOM is about 60 MHz for $P_{\mathrm{in}} > 2.18$ mW, reasonably close to the $f_{\mathrm{omo}}/2$. Consequently, the 1/2-harmonic locking occurs, and discrete frequency with integer multiple $f_{\mathrm{omo}}/2$ is present in the theoretical simulations (labelled region *III* in Supplementary Figure 8c] and experimental observations (shown in Supplementary Figure 3c). Since the SOM frequency varies with the change of driving conditions, other orders of frequency locking and different OMO sub-harmonic states can also be observed experimentally [Supplementary Figure 1 & 3] and theoretically [Supplementary Figure 5, 7 & 8c].

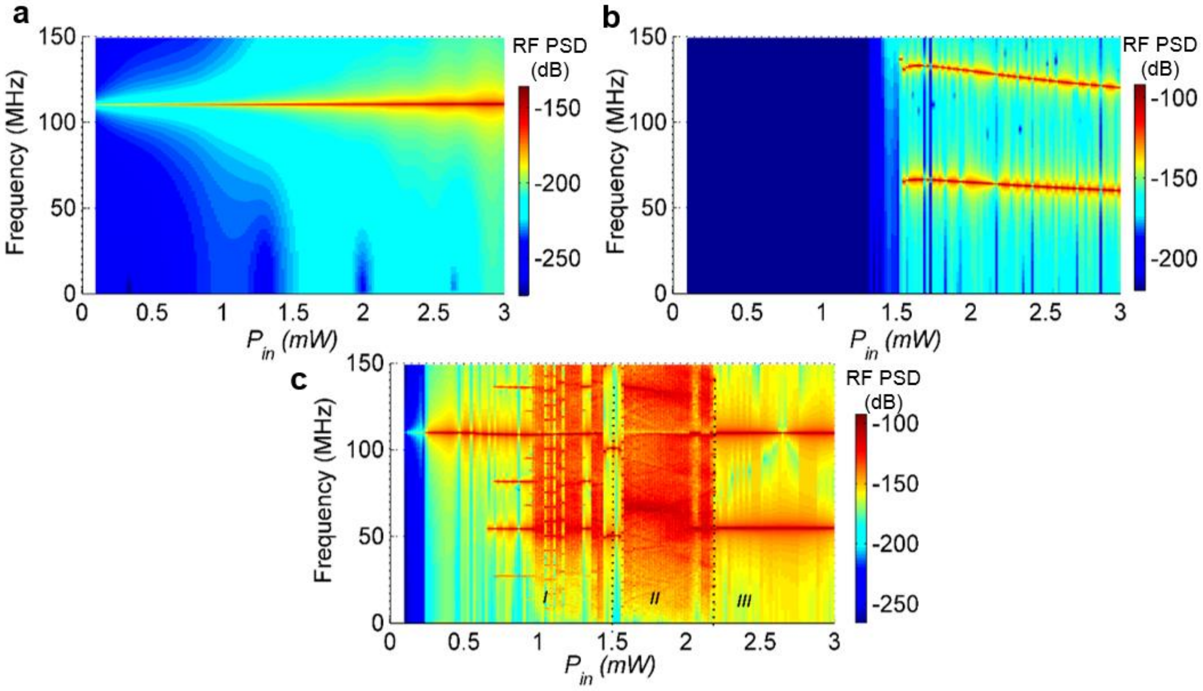

Supplementary Figure 8. **Investigations of the coupling between OMO and plasma.** (**a**), Modelled optomechanical oscillation (OMO) evolution under varying injection powers P_{in} without any photonic crystal cavity nonlinearities. (**b**), Modelled cavity evolution under varying injection powers P_{in} with cavity nonlinearity but without optomechanical mechanism by setting g_0 to 0. (**c**), Dynamical evolution of the recombined optomechanical photonic crystal cavity system under varying injection powers P_{in}, with g_0= 690 kHz and the cavity nonlinearity values shown in Supplementary Table 1. The three dynamical regions are labelled '*I*' (for P_{in} < 1.56 mW), '*II*' (for 1.56 < P_{in} < 2.18 mW) and '*III*' (for P_{in} > 2.18 mW), respectively.

Supplementary Note 7: Effects of the intracavity energy and drive power

Supplementary Figure 9a further illustrates the evolution curves of the OMO frequency and the two-photon-induced SOM frequency under different injection powers. Combining the dynamical evolution presented in Supplementary Figure 8c, there are three dynamical regions: *I* labels the bifurcation transition routes from pure OMO to $f_{omo}/2$, $f_{omo}/4$, then into the chaos region; *II* labels the chaos region; and *III* labels the $f_{omo}/2$ region. Firstly, in region *II*, the OMO couples strongly with SOM. The oscillation rhythm is disrupted and leads to the intricate chaotic states. In region *III*, the SOM frequency decreases to close to half of the OMO frequency. Meanwhile, the OMO becomes much stronger than that of region *I* and of region *II* according to the Supplementary Figure 8a. Then, frequency locking happens between OMO and SOM, and $f_{omo}/2$ is formed. Third, in the region *I*, there is no apparent SOM as illustrated in Supplementary Figure 8b. But in Supplementary Figure 8c, the system is unstable with a series of bifurcations and evolves gradually into the chaos state. As seen in Supplementary Figure 9b & 9c, one could find that the P_{in} already stimulates considerable N and ΔT in the region *I* in optomechanical photonic crystal cavity when the intracavity mode energy $|A|^2$ is relatively low as shown in Supplementary Figure 9d. These N and ΔT increase constantly along with the increase of P_{in}, also significantly destabilizing the OMO, and then leads to the bifurcation of the $f_{omo}/2$ state, $f_{omo}/4$ state, and even the chaos state. The above results illustrate that the large coupling between OMO and photonic crystal nonlinearities is key for the chaos generation.

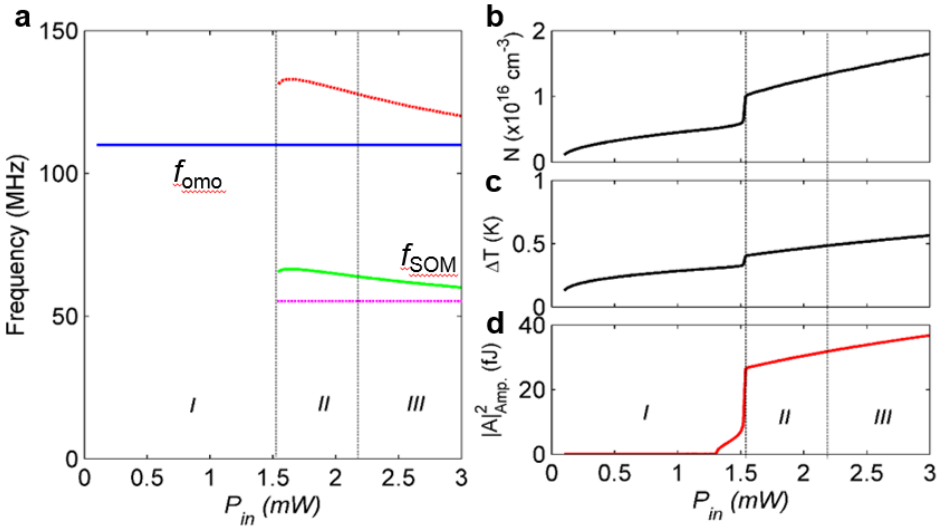

Supplementary Figure 9. **Evolution of the OMO, SOM and the PhC nonlinearities.** (**a**), Evolution of OMO frequency f_{omo} (blue line) and SOM frequency f_{SOM} (green line) and $f_{\mathrm{omo}}/2$ (purple dot line) and 2nd harmonics of f_{SOM} (red dot line) under varying injection powers P_{in}. (**b**) Corresponding evolution of intracavity free carrier density N. (**c**) Evolution of intracavity temperature variation ΔT. (**d**) Evolution of the amplification of the intracavity mode energy $|A|^2$. The three regions are labelled as '*I*' (for P_{in} < 1.56 mW), '*II*' (for 1.56 < P_{in} < 2.18 mW) and '*III*' (for P_{in} > 2.18 mW), respectively.

Supplementary References

1. Schreiber, T. Determination of the noise level of chaotic time series. *Phys. Rev. E* **48**, R13-R16 (1993).
2. Sprott J. C. *Chaos and Time Series Analysis* (Oxford University Press, 2003).
3. Brown, R., Bryant, P. & Abarbanel H. D. I. Computing the Lyapunov spectrum of a dynamical system from an observed time series. *Phys. Rev. A* **43**, 2787-2806 (1991).
4. Grassberger, P., & Procaccia, I. Characterization of strange attractors. *Phys. Rev. Lett.* **50**, 346-349 (1983).
5. Grassberger, P., & Procaccia, I. Estimation of the Kolmogorov entropy from a chaotic signal. *Phys. Rev. A* **28**, 2591-2593 (1983).
6. Grassberger, P., & Procaccia, I. Measuring the strangeness of strange attractors. *Physica D* **9**, 189-208 (1983).
7. Johnson, T. J., Borselli, M. & Painter, O. Self-induced optical modulation of the transmission through a high-*Q* silicon microdisk resonator. *Opt. Express* **14**, 817-831 (2006).
8. Aspelmeyer, M., Kippenberg, T. J. & Marquardt F. Cavity optomechanics. *Rev. Mod. Phys.* **86**, 1391-1452 (2014).
9. Yang, J. *et al.* Radio frequency regenerative oscillations in monolithic high-*Q/V* heterostructured photonic crystal cavities. *Appl. Phys. Lett.* **104**, 061104 (2014).
10. Li, T.-Y., & Yorke, J. A. Period three implies chaos. *The American Mathematical Monthly* **82**, 985-992 (1975).
11. Lorenz, E. N. Deterministic nonperiodic flow. *J. Atmos. Sci.* **20**, 130-141 (1963).
12. Haken, H. Analogy between higher instabilities in fluids and lasers. *Phys. Lett. A* **53**, 77-78 (1975).
13. Teitsworth, S. W., Westervelt, R. M., & Haller, E. E. Nonlinear oscillations and chaos in electrical breakdown in Ge. *Phys. Rev. Lett.* **51**, 825-828 (1983).
14. Held, G. A., Jeffries, C., & Haller, E. E. Observation of chaotic behavior in an electron-hole plasma in Ge. *Phys. Rev. Lett.* **52**, 1037-1080 (1984).
15. Gwinn, E. G., & Westervelt, R. M. Frequency locking, quasiperiodicity, and chaos in extrinsic Ge. *Phys. Rev. Lett.* **57**, 1060-1063 (1986).
16. Pecora, L. M., & Carroll, T. L. Synchronization in chaotic systems. *Phys. Rev. Lett.* **64**, 821-824 (1990).

17. Garcia-Ojalvo, J. & Roy, R. Spatiotemporal communication with synchronized optical chaos. *Phys. Rev. Lett.* **86**, 5204-5207 (2001).
18. VanWiggeren, G. D. & Roy, R. Communication with chaotic lasers. *Science* **279**, 1198-1200 (1998).
19. Poincaré, H. *Science and Method* (Courier Dover Publications, 2013).
20. Chen, Y. C., Winful, H. G. & Liu, J.-M. Subharmonic bifurcations and irregular pulsing behavior of modulated semiconductor lasers. *Appl. Phys. Lett.* **47**, 208-210 (1985).
21. Schreiber, T. Determination of the noise level of chaotic time series. *Phys. Rev. E* **48**, R13-R16 (1993)
22. Coffey, D. S. Self-organization, complexity and chaos: the new biology for medicine. *Nat. Med.* **4**, 882-885 (1998).
23. Peitgen, H.-O., Jürgens, H., Saupe, D. *Chaos and fractals: new frontiers of science* (Springer, 2004).
24. Zheng, J. *et al.* Feedback and harmonic locking of slot-type optomechanical oscillators to external low-noise reference clocks. *Appl. Phys. Lett.* **102**, 141117 (2013).
25. Sun, Y., & Sukhorukov, A. A. Chaotic oscillations of coupled nanobeam cavities with tailored optomechanical potentials. *Opt. Lett.* **39**, 3543-3546 (2014).
26. Ma, J. *et al.*Formation and manipulation of optomechanical chaos via a bichromatic driving. *Phys. Rev. A* **90**, 043839 (2014).
27. Sciamanna M. & Shore. K. A., Physics and applications of laser diode chaos, *Nature Photon.* **9**, 151-162 (2015).
28. Thompson, J. D. *et al.* Strong dispersive coupling of a high-finesse cavity to a micromechanical membrane. *Nature* **452**, 72-75 (2008).
29. Carmon, T., Rokhsari, H., Yang, L., Kippenberg, T. J. & Vahala, K. J. Temporal behavior of radiation-pressure-induced vibrations of an optical microcavity phonon mode. *Phys. Rev. Lett.* **94**, 223902 (2005).
30. Kippenberg, T. J., Rokhsari, H., Carmon, T., Scherer, A. & Vahala K. J. Analysis of radiation-pressure induced mechanical oscillation of an optical microcavity. *Phys. Rev. Lett.* **95**, 033901 (2005).
31. Haus, H. A., *Waves and fields in optoelectronics* (Prentice-Hall, 1984).
32. Yang, X. & Wong, C. W., Coupled-mode theory for stimulated Raman scattering in high-Q/V_m silicon photonic band gap defect cavity lasers. *Opt. Express* **15**, 4763-4780 (2007).
33. Hossein-Zadeh, M., & Vahala K. J. An optomechanical oscillator on a silicon chip. *IEEE J. Sel. Top. in Quant. Elect.* **16**, 276-287 (2010).
34. Abrams, D. M., Slawik, A. & Srinivasan K. Nonlinear oscillations and bifurcations in silicon photonic microresonators. *Phys. Rev. Lett.* **112**, 123901 (2014).
35. Ott, E. *Chaos in Dynamical Systems* (Cambridge University Press, 2002).

S36. Gavartin, E. *et al.* Optomechanical coupling in a two-dimensional photonic crystal defect cavity. *Phys. Rev. Lett.* **106**, 203902 (2011).

S37. Luan, X. *et al.* An integrated low phase noise radiation-pressure-driven optomechanical oscillator chipset. *Sci. Rep.* **4**, 6842 (2014).

S38. Buskirk, R. V., & Jeffries C. Observation of chaotic dynamics of coupled nonlinear oscillators. *Phys. Rev. A* **31**, 3332-3357 (1985).
S39. Karabalin, R. B., Cross, M. C. & Roukes M. L. Nonlinear dynamics and chaos in two coupled nanomechanical resonators. *Phys. Rev. B* **79**, 165309 (2009).
S40. Mancinelli, M., Borghi, M., Ramiro-Manzano, F., Fedeli, J. M. & Pavesi L. Chaotic dynamics in coupled resonator sequences. *Opt. Express* **22**, 14505-14516 (2014).
S41. Wurl, C., Alvermann, A. & Fehske H. Symmetry breaking oscillations in membrane optomechanics. arXiv:1609.05645v1.
S42. Akahane, Y., Asano, T., Song, B.-S. & Noda S. High-*Q* photonic nanocavity in a two-dimensional photonic crystal. *Nature* **425**, 944-947 (2003).
S43. Gilbert T. & Gammon, R. Stable oscillations and Devil's staircase in the Van der Pol oscillator. *Int. J. Bifurcation Chaos* **10**, 155-164 (2000).
S44. Lin F. Y. & Liu, J. M. Harmonic frequency locking in a semiconductor laser with delayed negative optoelectronic feedback. *Appl. Phys. Lett.* **81**, 3128-3130 (2002).
